# Irrigation of the Intestine in Dysentery

**Published:** 1890-11

**Authors:** 


IRRIGATION OF THE INTESTINE IN DYSENTERY.—Dr. H. A Fairbairn (*Brooklgn Medical Journal,* October, 1890) gives unqualified praise to the treatment of dysentery by irrigation of the large intestine. To be of use, the enemata should be large and repeated every two, three, or four hours. The quantity of water should be, for an adult, about three or four pints, at a temperature of from 100 to 105 degrees. If possible the water should be distilled, or at least boiled.

Dr. Fairbairn has not found it necessary to use any medicament in the water. As to the instrument used, he prefers a handball-syringe, believing that the pressure from a fountain-syringe is dangerous. The author also suggests that in chronic dysentery the walls of the bowel may be so much weakened that unless the pressure is very slight rupture may occur.—*Medical News.*

